# Short- and long-term efficacy evaluation of high-intensity focused ultrasound ablation and uterine artery embolization in the treatment of cesarean scar pregnancy (CSP) I and II: a retrospective cohort study

**DOI:** 10.3389/fmed.2026.1805049

**Published:** 2026-03-27

**Authors:** Lingna Huang, Bo Long, Fengning Lin, Wen Ling, Liping Huang, Xiumei Xiong

**Affiliations:** 1 Department of Gynecology and Obstetrics, Fujian Maternity and Child Health Hospital, College of Clinical Medicine for Obstetrics, Gynecology, and Pediatrics, Fujian Medical University, Fuzhou, Fujian, China; 2 Department of Anesthesiology, The Second Affiliated Hospital of Fujian University of Traditional Chinese Medicine, Fuzhou, Fujian, China; 3 Department of Ultrasound, Fujian Maternity and Child Health Hospital, College of Clinical Medicine for Obstetrics, Gynecology, and Pediatrics, Fujian Medical University, Fuzhou, Fujian, China

**Keywords:** cesarean scar pregnancy, high-intensity focused ultrasound, minimally invasive treatment, reproductive outcomes, uterine artery embolization

## Abstract

**Objectives:**

To retrospectively analyze and compare the safety, effectiveness, and long-term pregnancy outcomes of high-intensity focused ultrasound (HIFU) versus uterine artery embolization (UAE), both followed by ultrasound-guided dilatation and curettage (USg-D&C), in treating cesarean scar pregnancy (CSP) type I and II, offering clinical insights.

**Design:**

A retrospective cohort study (Fujian Maternal and Child Health Hospital).

**Setting:**

University hospital.

**Patients:**

From June 2021 to December 2024, a total of 84 patients were included, all diagnosed with CSP I or II and treated by high-intensity focused ultrasound (HIFU) or uterine artery embolization (UAE), followed by USg-D&C.

**Interventions:**

None.

**Measurements and main results:**

Follow-up of the reproductive outcomes ended in December 2024, 40 patients received HIFU ablation and 44 patients received UAE both followed by USg-D&C, involved in the study.

**Results:**

Baseline characteristics were comparable between the two groups (all *p* > 0.05). The HIFU group had a significantly shorter hospital stay than the UAE group (4.7 ± 1.8 days vs. 5.9 ± 2.7 days, *p* = 0.042) and a lower incidence of reduced menstrual flow (25.0% vs. 47.7%, *p* = 0.002). No significant differences were found in intraoperative blood loss, operation time, β-hCG normalization time, or menstrual recovery time (all *p* > 0.05). The intrauterine pregnancy rate was 66.7% in the HIFU group and 47.4% in the UAE group (*p* = 0.259), but the HIFU group had a significantly shorter time from treatment to intrauterine pregnancy (18.1 ± 2.2 months vs. 22.7 ± 1.7 months, *p* = 0.019) and time from conception attempt to pregnancy (9.5 ± 1.3 months vs. 16.6 ± 2.1 months, *p* = 0.013).

**Conclusion:**

Compared with UAE, HIFU in the treatment of CSP type I and type II results in shorter hospital stays for patients, milder effects on menstrual volume, and shorter intervals to pregnancy and conception preparation durations. However, no statistically significant difference was noted in the long-term intrauterine pregnancy rate between the two groups. Future research should prioritize high-quality randomized controlled trials with prospective design, large sample sizes, and multi-center settings.

## Introduction

Cesarean scar pregnancy (CSP) is defined as an ectopic pregnancy wherein the gestational sac implants at the site of the uterine incision from a previous cesarean section. This condition represents a potential long-term complication following cesarean delivery and constitutes approximately 6.1% of all ectopic pregnancies post-cesarean section (1). CSP is associated with significant maternal risks, including uterine rupture and severe vaginal hemorrhage, which may result in mortality (2). Consequently, the primary objective of CSP management is the prompt termination of the pregnancy to prevent the onset of severe complications such as uterine rupture and extensive bleeding. Uterine artery embolization (UAE) in conjunction with ultrasound-guided dilation and curettage (USg-D&C) is regarded as a minimally invasive treatment modality for CSP that facilitates rapid recovery while optimizing the preservation of patient fertility (3). Nonetheless, recent research indicates that UAE may adversely impact endometrial and ovarian function, potentially influencing future pregnancies (4). With advancements in science and technology, high-intensity focused ultrasound (HIFU) ablation combined with USg-D&C has been widely used for the treatment of CSP in type I and type II uterine scar pregnancies, with its safety and effectiveness preliminarily confirmed (5). However, there is still limited follow-up data on pregnancy outcomes after treatment. This study seeks to conduct a retrospective analysis to compare the safety and efficacy of HIFU ablation combined with USg-D&C versus UAE combined with USg-D&C in the treatment of CSP type I and II. Additionally, it aims to evaluate their impact on long-term reproductive and pregnancy outcomes in patients, thereby offering a reference for the clinical diagnosis and management of CSP type I and II.

## Materials and methods

### Patients

**Figure 1:**
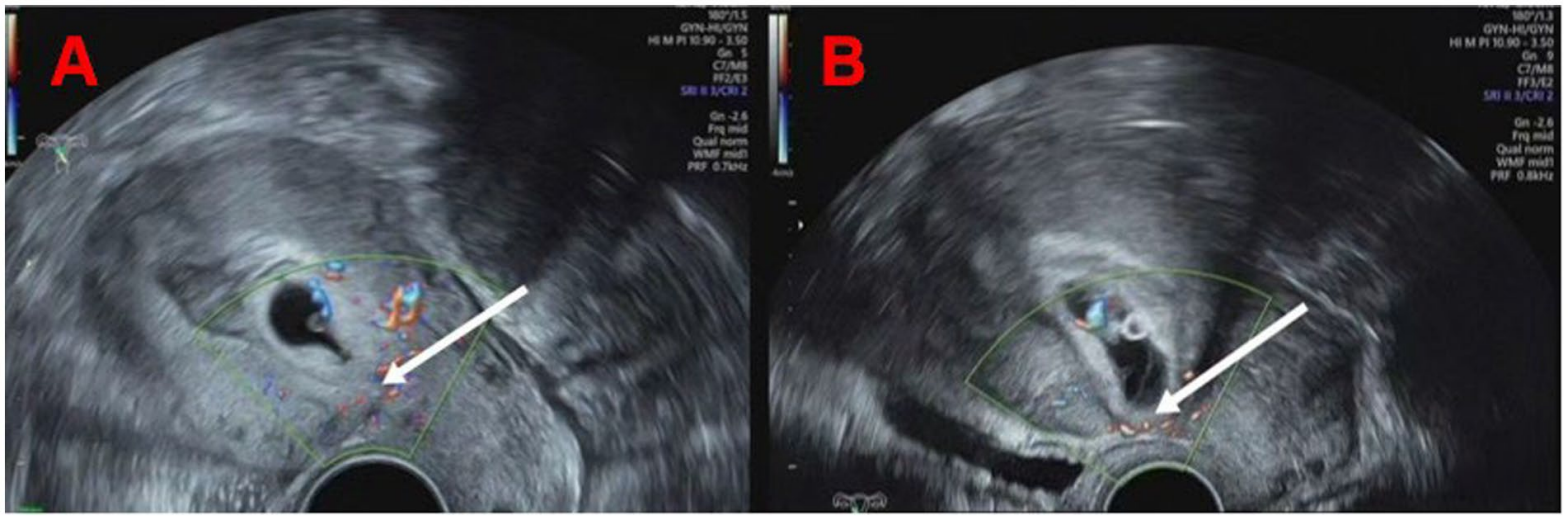
Typical color Doppler ultrasound images of type I and II CSP.

This study conducted a retrospective analysis of 111 patients diagnosed with CSP type I and II who received treatment involving HIFU ablation in conjunction with USg-D&C or UAE combined with USg-D&C at the Gynecology Department of Fujian Maternal and Child Health Hospital from June 2021 to December 2024. Prior to and following treatment, all participants underwent standard vaginal sagittal and transverse color Doppler ultrasound or magnetic resonance imaging (MRI). Two independent researchers (Bo Long and Fengning Lin) retrospectively classified the cases by evaluating static images derived from CSP medical history records, ultrasound examinations, and MRI scans. CSP classification was based on color Doppler ultrasound or MRI, i.e., Type I: the gestational sac is partially located at the scar site, and the uterine muscle layer between the gestational sac and the bladder becomes thinner, with a thickness of ≥ 3 mm; Type II: The gestational sac is partially located at the scar site, and the uterine muscle layer between the gestational sac and the bladder becomes thinner, with a thickness of less than 3 mm (6) (Figure 1). The selection of HIFU combined with USg-D&C or UAE combined with USg-D&C for patients with type I and type II CSP was non-randomized, and the treatment strategy was determined based on a comprehensive assessment of the patients’ clinical characteristics, individual conditions, and the professional judgment of the treating physicians, which is consistent with the clinical practice of retrospective studies on CSP treatment. Patient demographics and clinical data were also recorded, including age, BMI, number of previous cesarean sections, preoperative blood β-hCG levels, gestational age, and clinical symptoms.

The inclusion criteria for the study are as follows: (1) aged over 20 years; (2) history of cesarean section; (3) presenting with amenorrhea and positive urine pregnancy test; (4) empty uterine cavity and cervical canal; (5) gestational sac located in the anterior lower wall of the uterus; (6) ultrasound and MRI findings meeting the diagnostic criteria for type I and II CSP. Exclusion criteria: (1) unstable vital signs; (2) previous treatment for CSP; (3) uncertain CSP diagnosis; (4) history of trophoblastic disease and cardiovascular diseases; (5) direct surgical treatment; (6) incomplete clinical or follow-up data.

This study adhered to the ethical principles outlined in the 1975 Helsinki Declaration, as revised in 2013. Approval was obtained from the ethics committee of Fujian Maternal and Child Health Hospital (Approval number: 2025KY111). Written informed consent was obtained from all participants prior to any study-related procedures.

### Procedure for UAE

UAE treatment was completed in the interventional surgery room. The main treatment procedures were performed by attending physicians or higher in the interventional department. The UAE procedure involved routine disinfection and draping, local anesthesia with 2% lidocaine, Seldinger technique for puncturing the right femoral artery to place a vascular sheath, guiding a 4F Cobra catheter into the left and right internal iliac arteries, injecting 8 mL of iodixanol contrast at a flow rate of 3 mL/s, selectively catheterizing the left and right uterine arteries with microcatheters, injecting MTX 30 mg and 560–710 μm gelatin sponge particles, and performing a repeat angiography showing stasis of blood flow in the distal branches of the bilateral uterine arteries and significant reduction in main trunk blood flow.

### HIFU treatment

HIFU treatment is completed in the HIFU treatment room. The main treatment procedures are carried out by physicians with senior professional titles and above. The specific HIFU treatment process: HIFU treatment is performed using a focused ultrasound tumor treatment system (model PRO300; Shenzhen Pro Medical Co., Ltd.). Prior to HIFU treatment, all patients are required to shave the front abdominal hair from the umbilicus to the pubic symphysis, and insert a urinary catheter to control bladder capacity.

All patients received a single session of HIFU ablation therapy. The procedure was performed under conscious sedation (5 mg of hydrocodone administered intravenously over 2 min, followed by 5 mg of hydrocodone in 25 mL of normal saline at a rate of 6–8 mL/h). Patients were positioned supine on the HIFU system, with the transducer in contact with the anterior abdominal wall covered with degassed water in a sealed container. Real-time ultrasound was used to locate the gestational sac target area and monitor treatment response. A sagittal ultrasound scanning mode was used to divide the gestational sac into 2 mm thick slices for treatment planning, starting from the innermost slice. The acoustic power output ranged from 280–320 W, and ultrasound irradiation was terminated when color Doppler ultrasound showed disappearance of blood flow signals in the pregnancy tissue or when grayscale changes appeared in the target area.

### Ultrasound-guided dilation and curettage (USg-D&C)

After UAE or HIFU ablation, a curettage is performed under general anesthesia within 72 h. The lithotomy position is taken, and the pregnancy tissue in the uterine muscle defect is located by ultrasound. A 6-7 mm suction tube is gently inserted into the uterine cavity, with a negative pressure set at 400 mmHg. The operator moves the tube back and forth, gently separating and suctioning out the pregnancy tissue from the cesarean scar (7). The treatment procedure is carried out by one doctor performing ultrasound-guided positioning to show the location of the pregnancy tissue, while another gynecologist of senior title or above performs the hysteroscopy surgery.

### Follow-up

After discharge, beta-human chorionic gonadotropin (β-hcg) levels should be checked weekly until they decrease to normal range. A gynecological transvaginal ultrasound should be done 1 month post-surgery, with the patient advised to monitor for vaginal bleeding, recovery of menstruation, and menstrual flow. Follow-up calls every 6 months will assess the patient’s general condition, changes in menstrual cycle and flow, and fertility needs. For those seeking pregnancy, record their preconception period and subsequent pregnancy outcomes.

### Observation indicators and measures

Main outcome measures: (1) Treatment success rate: Successful completion of uterine evacuation surgery without complications, clear uterine cavity on ultrasound with no obvious tissue residue. The absence of major intraoperative bleeding and no additional surgical treatment required. Weekly monitoring of β-hcg levels post-operatively, with a decrease of <15% prompting consideration of additional medical treatment (e.g., mifepristone) or a second surgery. A second surgery is defined as treatment failure. (2) Rate of subsequent intrauterine pregnancy: For individuals desiring fertility or those not practicing effective contraception, confirmation of a subsequent intrauterine pregnancy on ultrasound is recorded as a subsequent pregnancy.

Secondary measures: Intraoperative blood loss during curettage surgery; duration of curettage surgery; time required for postoperative β-HCG levels to normalize; time to postoperative menstrual recovery and assessment of menstrual volume. The definition of reduced menstrual flow is determined by the number of sanitary pads used: if the number of pads replaced (or tampons used) that become soaked each day decreases from the pre-treatment (mean ± standard deviation) to the post-treatment (mean ± standard deviation), and the total usage reduction is ≥30%, then it is considered as reduced menstrual flow.

### Statistical analysis

Statistical analyses were performed with SPSS 29.0 software. Continuous variables with normal distribution are presented as mean ± standard deviation, and non-normal discrete variables as median and interquartile range (IQR). Independent-samples *t*-test was used for normally distributed continuous variables with homogeneous variance, and Mann–Whitney *U* test for non-conforming variables. Categorical data were analyzed by chi-square test. Kaplan–Meier survival curves with the Breslow test were used to assess cumulative pregnancy rate and time to intrauterine pregnancy after CSP treatment. All tests were two-tailed, with *p* < 0.05 considered statistically significant.

A post-hoc power analysis was conducted via G*Power 3.1 for the key reproductive outcome (intrauterine pregnancy rate). For the two-tailed Chi-square test of independence (*α* = 0.05), with conception-attempting group sizes of 15 (HIFU) and 19 (UAE), and an observed effect size (Cohen’s *h*) of 0.41, the resultant statistical power was 0.32, below the conventional 0.80 threshold. This analysis quantified the limitation of small sample size in detecting potential statistical differences in intrauterine pregnancy rates between groups.

## Results

### Follow-up results

**Figure 2:**
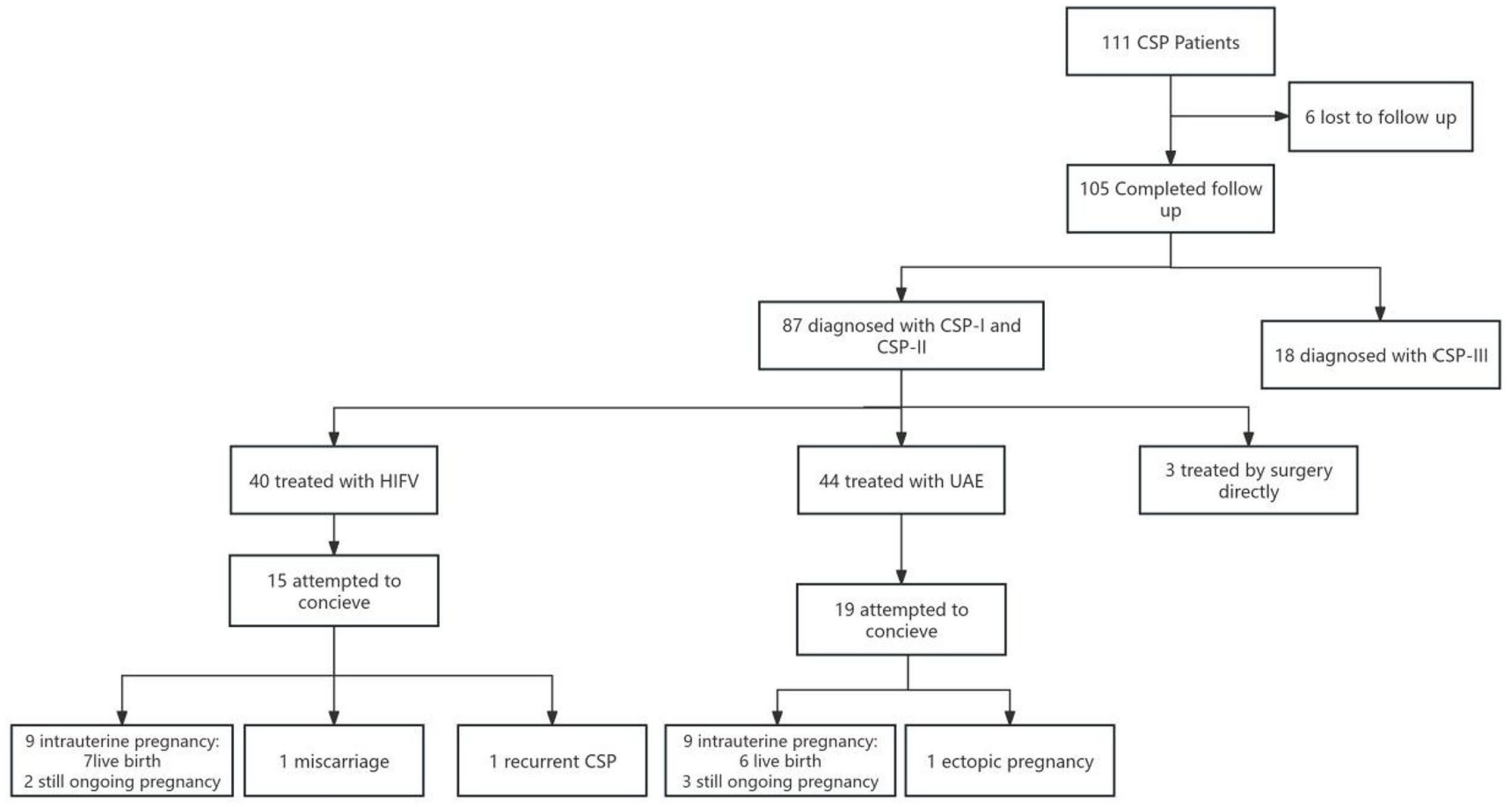
Follow-up results.

Between June 2021 to June 2024, 111 women with a CSP were diagnosed in our hospital, 6 were lost to follow-up due to loss of contact. Among the 105 patients who completed follow-up, 87 patients were diagnosed with type I or II CSP, and 3 were excluded following directly curettage surgery. A total of 84 patients were ultimately included in the follow-up study, including 40 who received HIFU ablation combined with USg-D&C treatment and 44 who received UAE combined with USg-D&C. The follow-up period ranged from 1 to 4.5 years. 15 patients (37.5%) in the HIFU treatment group had a need for reproduction, while 19 patients (43.18%) in the UAE treatment group had a need for reproduction. During the study period, 10 patients in the HIFU group had another intrauterine pregnancy, 1 case of recurrent CSP, 9 patients in the UAE group had an intrauterine pregnancy, and 1 case had an ectopic pregnancy (Figure 2). In patients with no desire for future fertility, an additional 2 cases of unintended pregnancy without contraception were recorded in the high-intensity focused ultrasound (HIFU) group, versus 3 such cases in the uterine artery embolization (UAE) group.

### Baseline characteristics

The average ages of the HIFU group and UAE group were 34.13 ± 4.85 years and 34.24 ± 5.07 years, respectively, with corresponding body mass indices (BMI) of 22.1 ± 3.7 kg/m^2^ and 23.5 ± 3.9 kg/m^2^, respectively. There was no significant difference between the groups. In the HIFU group, 18 cases were classified as type I CSP and 22 cases were classified as type II CSP; In the UAE group, 21 cases were type I CSP and 23 cases were type II CSP; There were no statistically significant differences between the two groups in terms of the number of previous cesarean sections, the time between CSP and the previous cesarean section, pre-treatment HCG levels, gestational weeks, pre-treatment muscle layer thickness, and whether the embryo had heartbeats before treatment; There was no statistically significant difference in the number of previous cesarean sections, clinical symptoms, and whether the embryos had heartbeats before treatment between the two groups; there were 10, 20, 4, and 6 asymptomatic, painless vaginal bleeding, lower abdominal pain and abdominal pain with vaginal bleeding cases, respectively, in HIFU Group. In the UAE group, there were15, 22, 3, and 4 asymptomatic, painless vaginal bleeding, lower abdominal pain and abdominal pain with vaginal bleeding cases respectively. There was no statistical difference in the clinical symptoms of two groups (Table 1).

**Table 1:** The baseline characteristics of patients with HIFU and UAE group.

Characteristics	HIFU	UAE	*p*
Material age (y)	34.13 ± 4.85	34.24 ± 5.07	0.924
Body mass index (kg/m^2^)	22.1 ± 3.7	23.5 ± 3.9	0.837
Previous cesarean section deliveries			0.65
1	20	21	
2	19	20	
≥3	1	3	
Time between CSP and the previous cesarean section (years)	5.6 ± 3.7	6.3 ± 4.2	0.216
CSP type			0.802
I	18	21	
II	22	23	
HCG level (U/L)	25761.92 (9132–68481)	19349.5 (7479–74702)	0.391
Pregnancy duration (d)	45.2 ± 10.1	50.1 ± 15.3	0.078
Symptom			0.69
None	10	15	
Vaginal bleeding	20	22	
Abdominal pain	4	3	
Vaginal bleeding and abdominal pain	6	4	
Cardiac activity			0.741
Yes	18	20	
No	22	24	
Residual myometrium thickness (mm)	0.23 ± 0.12	0.20 ± 0.15	0.367

### Short-term outcomes

Among all the patients with CSP type I and II who received HIFU or UAE treatment included in the study, only one patient in the UAE group had vaginal bleeding greater than 400 mL during curettage surgery. Despite the application of active conservative measures, such as balloon compression, to control the hemorrhage, these efforts proved ineffective, necessitating conversion to laparotomy. All other patients successfully completed the curettage procedure. The median intraoperative bleeding volume was 10 mL (IQR: 5–50 mL) in the HIFU group and 5 mL (IQR: 2–20 mL) in the UAE group, with no statistically significant difference observed [*p* = 0.083; 95% CI: (−2.1, 18.7)]. The median duration of curettage surgery was 20 min (IQR: 10–40 min) in the HIFU group and 15 min (IQR: 12–20 min) in the UAE group, also showing no statistically significant difference [*U* = 635.5, *p* = 0.124; 95% CI: (−5.3, 12.8)]. The average length of hospital stay was significantly different between the groups, with the HIFU group averaging 4.7 ± 1.8 days and the UAE group averaging 5.9 ± 2.7 days (*p* = 0.042); After discharge, the time for β-hCG levels to normalize was 2.9 ± 1.3 weeks and the time for menstrual recovery was 1.3 ± 0.7 months in the HIFU group, while in the UAE group, the corresponding times were 3.1 ± 1.4 weeks and 1.3 ± 0.5 months, with no statistically significant differences. Following treatment, 10 patients (25.0%) in the HIFU group experienced menstrual reduction, compared to 21 patients (47.7%) in the UAE group, showing a statistically significant difference (*p* = 0.002) (Table 2).

**Table 2:** The short-outcome of patients with HIFU and UAE group.

Characteristics	HIFU	UAE	*p*
Success of therapy	40	43	
Blood loss volume (mL)	10 (5–50)	5 (2–20)	0.083
Operation time (min)	20 (10–40)	15 (12–20)	0.124
Time of hospital stay (d)	4.7 ± 1.8	5.9 ± 2.7	0.042
Time for β-HCG return to normalization (wk)	2.9 ± 1.3	3.1 ± 1.4	0.521
Menstrual recovery time (months)	1.3 ± 0.7	1.3 ± 0.5	0.792
Volume of menstrual			0.002
No significantchanges	30	22	
Reduce	10	21	
Increase	0	1	

### Reproductive outcomes

**Figure 3:**
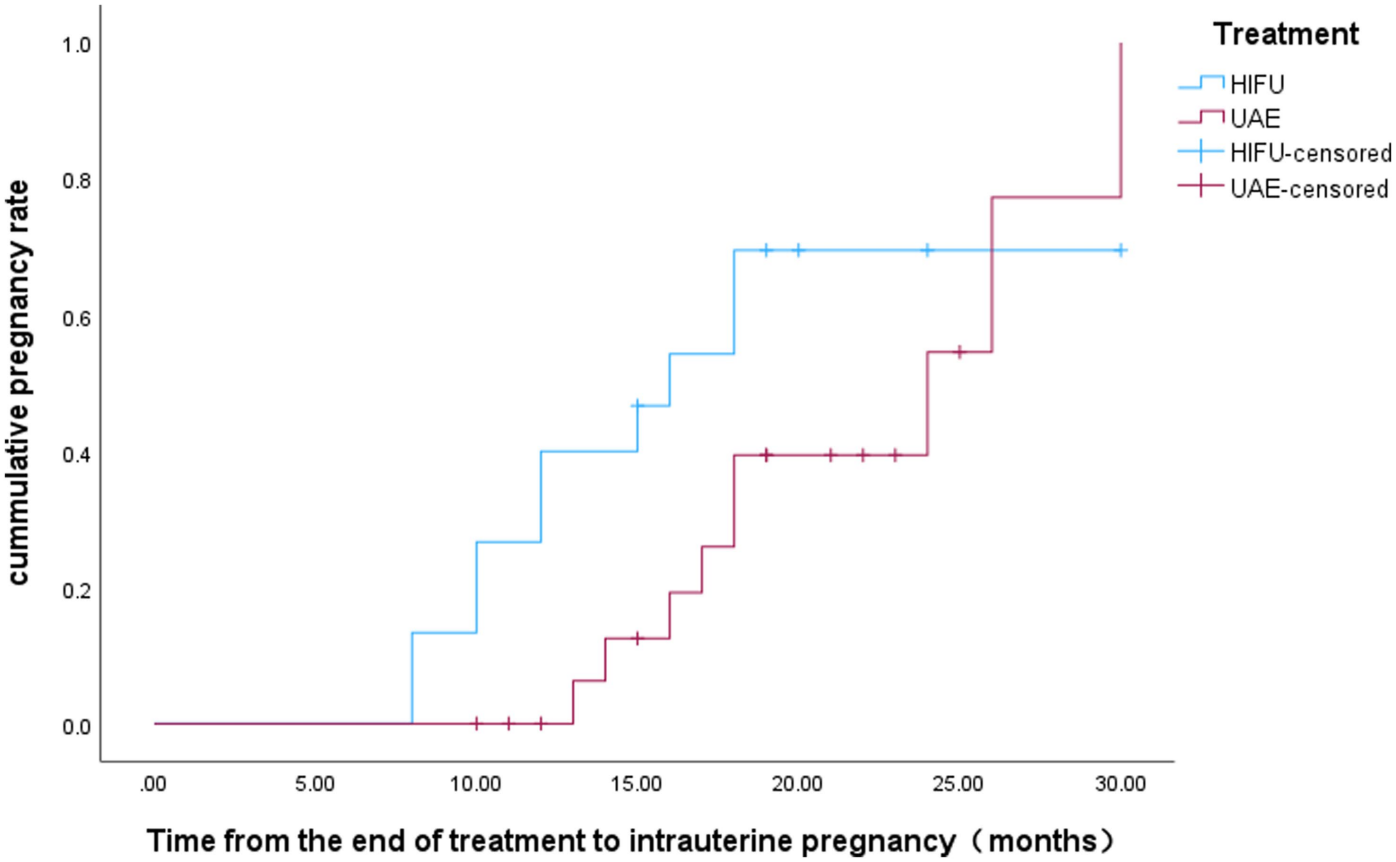
Kaplan-Meier plot for time interval between the end treatment of CSP and subsequent intrauterine pregnancy in both groups.

**Figure 4:**
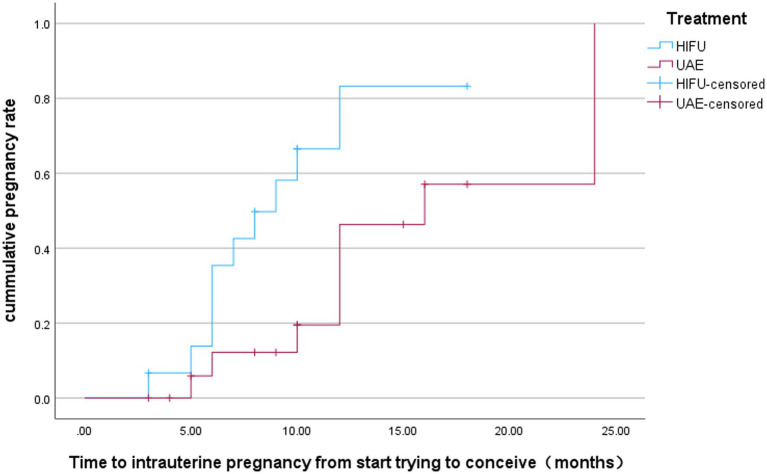
Kaplan-Meier plot for time interval between start trying to conceive and subsequent intrauterine pregnancy in both groups.

There were 15 women in the HIFU group who attempted to conceive after surgery. As of the last follow-up of this study (December 2025), 10 cases (66.7%) had successful intrauterine pregnancies, including 5 cases of successful delivery, 4 cases of still ongoing pregnancy, 1 case of spontaneous miscarriage in early pregnancy, and 1 case of recurrent CSP. In the UAE treatment group, 19 patients attempted conception postoperatively, with 9 (47.4%) achieving successful intrauterine pregnancy. Of these 9 cases, 6 have delivered and 3 are ongoing pregnancies; 1 additional case was diagnosed with ectopic pregnancy. No statistically significant difference was observed in the rate of postoperative intrauterine pregnancy between the two groups (*p* = 0.259) (Table 3). The mean interval from CSP treatment to subsequent intrauterine pregnancy was 18.105 ± 2.228 months in the HIFU group versus 22.688 ± 1.726 months in the UAE group, with a statistically significant difference [*p* = 0.019; 95% CI: (10, 24.4)] (Figure 3). For conception preparation duration, the mean was 9.545 ± 1.342 months in the HIFU group versus 16.648 ± 2.056 months in the UAE group [*p* = 0.013; 95% CI: (5.3, 18.7)] (Figure 4).

**Table 3:** The reproductive outcomes of patients with HIFU and UAE group.

Characteristics	HIFU	UAE	*p*
Attempted to conceive	15	19	0.596
Intrauterine pregnancy	10	7	0.084
Live birth	5	5	
Still ongoing pregnancy	4	2	
Miscarriage	1	0	
Ectopic pregnancy	0	1	
Recurrent CSP	1	0	
The duration of intrauterine pregnancy from the end of the last CSP treatment (mo)	18.1 ± 2.2	22.7 ± 1.7	0.019
Time to intrauterine pregnancy from start trying to conceive (mo)	9.5 ± 1.3	16.6 ± 2.1	0.013

## Discussion

The marked rise in cesarean section rates has resulted in a corresponding increase in the prevalence of uterine scars and associated complications in subsequent pregnancies, with cesarean scar pregnancy (CSP) being a notable long-term complication (8). In 2000, Vial et al. (9) were the first to document cases of pregnancies occurring at the scar site on the anterior uterine wall, after which scholarly reports concerning CSP have proliferated significantly. CSP is associated with an elevated risk of severe complications, including placental implantation abnormalities, uterine rupture, hemorrhagic shock, and the potential necessity for total hysterectomy during pregnancy, thereby posing a substantial threat to women’s reproductive health and, in some cases, their lives (10). The prevailing diagnostic and therapeutic approach for first trimester CSP involves the prompt termination of the pregnancy and removal of the gestational material upon diagnosis (11). Nonetheless, with varying types of CSP, determining a safe, effective, and minimally invasive treatment strategy that optimally preserves fertility remains a clinical challenge, particularly for patients who have reproductive aspirations.

Owing to its advantages of minimal trauma and reliable hemostasis, UAE is extensively utilized as an adjuvant therapy for a variety of gynecological and obstetric conditions. Its efficacy as an adjunctive treatment prior to cesarean scar pregnancy (CSP) curettage has been well-documented (3, 12). The therapeutic mechanism of UAE involves the embolization of the uterine artery to obstruct the blood supply to the embryo (3), thereby reducing intraoperative hemorrhage during the curettage. Nevertheless, some studies have indicated that the incidence of postoperative complications associated with UAE ranges from 8.0 to 51.7%, with early complications such as fever, lower abdominal pain, and bruising at the puncture site being more prevalent (13). As the condition advances, some patients may experience a reduction in menstrual flow, and in severe cases, secondary intrauterine adhesions (with an incidence rate of 30%) and amenorrhea (with an incidence rate of 1 to 15%) may occur, significantly impacting the patient’s reproductive function (14). The HIFU group had a statistically significantly shorter hospital stay than the UAE group (4.7 ± 1.8 days vs. 5.9 ± 2.7 days, *p* = 0.042), and this 1.2-day mean difference has notable clinical significance for CSP management. The shorter hospital stay in the HIFU group is inherently linked to the minimally invasive and non-invasive characteristics of HIFU ablation: unlike UAE, which involves femoral artery puncture, vascular catheterization and embolization, HIFU is a non-invasive procedure without surgical incisions or vascular interventional trauma, resulting in fewer postoperative adverse reactions (e.g., embolism syndrome, puncture site pain) that require in-hospital observation and management. On the other hand, our findings demonstrate that the UAE group exhibits a higher incidence of postoperative menstrual reduction compared to the HIFU group. This phenomenon may be due to the UAE procedure simultaneously obstructing the anastomotic branches of the ovarian artery, potentially leading to ovarian dysfunction and impaired endometrial proliferation (3). In a study by Tulandi et al. (15), hormone levels (FSH and E2), antral follicle count, ovarian volume, and blood flow were measured before and after UAE. The findings revealed a significant increase in FSH levels post-UAE, suggesting a reduction in ovarian reserve capacity. Consequently, it is advised that patients with fertility needs exercise caution when opting for this treatment (16). Tropean et al. (17) reported a case of a 44-year-old patient who experienced amenorrhea after UAE. Subsequent examinations suggested that the procedure impaired endometrial hyperplasia function, resulting in amenorrhea (15). However, many scholars posit that uterine artery embolization (UAE) for gynecological disorders does not compromise ovarian function, or that any reduction in ovarian function is reversible (17, 18). This reversibility may be attributed to the reconstruction of collateral circulation and the self-repair mechanisms of ovarian tissue (19). Our findings indicated that for patients desiring subsequent fertility, despite longer intervals to pregnancy and conception preparation durations in the UAE group, no statistically significant difference was detected in the long-term intrauterine pregnancy rate between the two groups. This observation may be attributed to the transient decline in ovarian function, which exerts no marked impact on subsequent intrauterine pregnancy rates following ovarian self-recovery via collateral circulation reconstruction. However, further high-quality studies are warranted to clarify the mechanisms underlying the long-term effects of UAE on ovarian function, thereby optimizing treatment strategies.

With the advancement of minimally invasive surgical techniques and the enhancement of patients’ quality of life, HIFU has gained widespread application in the treatment of CSP due to its non-invasive nature, high reproducibility, rapid postoperative recovery, and minimal complications, demonstrating favorable therapeutic outcomes (7, 20). In comparison to UAE, HIFU offers precise control over treatment parameters such as power, range, and duration. It effectively targets uterine scar tissue, facilitating the destruction of the gestational sac and surrounding blood vessels through thermal ablation. The use of real-time ultrasound monitoring during the procedure minimizes the risk of damage to the endometrium and ovaries, with exerting minimal impact on ovarian blood supply and endocrine function. Numerous studies indicate that HIFU treatment has negligible effects on ovarian function and does not elevate the risk of infertility or adverse pregnancy outcomes (21). Our findings demonstrated that in the HIFU group, not only was the incidence of postoperative menorrhagia reduction significantly lower than that in the UAE group, but the subsequent intervals to intrauterine pregnancy and conception preparation durations were also notably shorter. A meta-analysis of 21 studies involving 29,661 patients by Ji et al. (22) yielded consistent results: HIFU is associated with a higher reintervention rate, yet it offers a higher pregnancy rate and a shorter interval to pregnancy. Thus, we conclude that HIFU exerts a relatively milder impact on ovarian function, which is conducive to the recovery of postoperative fertility in patients.

Current research predominantly indicates that HIFU may be a preferable treatment option for patients with CSP who have fertility needs (22, 23). However, HIFU treatment presents certain limitations, particularly in the management of CSP type III, necessitating meticulous patient selection. This is due to the fact that the gestational sac in CSP III frequently invades the deeper layers of the uterine muscle and may even penetrate the uterine wall, coming into proximity with the bladder or pelvic blood vessels. HIFU operates by using focused ultrasound to generate heat and ablate lesions, but the extended energy transfer path can make it challenging to accurately target deep lesions, posing a risk of damage to adjacent organs such as the bladder. Furthermore, the blood supply to the gestational sac in CSP III is typically more abundant, and the thermal effect of HIFU may not effectively occlude large blood vessels, thereby increasing the risk of postoperative hemorrhage. Additionally, the high temperatures generated by HIFU may induce vasodilation, which could further exacerbate bleeding. Consequently, current clinical guidelines and research advocate for surgical or interventional treatment as the preferred approach for CSP III, whereas HIFU is more appropriate for specific cases of CSP I or II (24, 25). In clinical practice, it is imperative to conduct a comprehensive assessment of the patient’s condition and potential reproductive outcomes to formulate individualized management strategies, especially for patients with fertility needs. It is advisable to prioritize treatment methods that preserve ovarian blood supply under the premise of safety, and ovarian function indicators should be routinely monitored postoperatively.

The limitations of this study include: Post-hoc power analysis (G*Power 3.1) for intrauterine pregnancy rate comparison (HIFU: *n* = 15, UAE: *n* = 19) showed a statistical power of merely 0.32 (Cohen’s *h* = 0.41, *α* = 0.05, two-tailed), well below the 0.80 threshold, which was due to the small sample of conception-attempting patients caused by CSP-related pregnancy anxiety and patients in the recommended 6–12-month contraceptive period during the 1–4.5-year follow-up, limiting the detection of potential statistical differences in pregnancy rates despite clinical trends. Additionally, the retrospective single-center design, small overall sample and lack of objective ovarian function markers (AMH, FSH) for postoperative assessment compromised the study’s statistical rigor and mechanistic inferences for ovarian function impacts. Inherent selection bias existed as treatment allocation (HIFU/UAE) relied on clinical decisions and patient choice rather than randomization; though baseline characteristics were comparable (all *p* > 0.05), unmeasured confounders might still affect outcomes. Information bias was also present: menstrual flow reduction was subjectively assessed via self-reported sanitary pad usage without objective quantification, and the time from conception attempt to pregnancy was based on patient recall, potentially introducing recall bias.

## Data Availability

The original contributions presented in the study are included in the article/supplementary material, further inquiries can be directed to the corresponding author.
